# Ion therapy within the trimodal management of superior sulcus tumors: the INKA trial

**DOI:** 10.1186/s12885-015-1163-7

**Published:** 2015-03-28

**Authors:** Henrik Hauswald, Stefan Rieken, Hendrik C Dienemann, Michael Thomas, Meinhard Kieser, Jürgen Debus, Klaus Herfarth

**Affiliations:** 1 Department of Radiation Oncology, University Hospital of Heidelberg, Heidelberg, Germany; 2 Heidelberg Institute of Radiation Oncology (HIRO), Heidelberg, Germany; 3 Department of Thoracic Surgery, Thoraxklinik, University Hospital of Heidelberg, Heidelberg, Germany; 4 Department of Thoracic Oncology/Internal Medicine, Thoraxklinik, Translational Lung Research Center Heidelberg (TLRC-H), Member of the German Center for Lung Research (DZL), University Hospital of Heidelberg, Heidelberg, Germany; 5 Institute for Medical Biometry and Informatics, University of Heidelberg, Heidelberg, Germany

**Keywords:** Superior sulcus tumors, Pancoast tumor, Ion beam therapy, Heavy ion therapy, Irradiation, Lung cancer

## Abstract

**Background:**

The standard trimodal treatment concept in locally advanced and non-metastasized non-small-cell superior sulcus tumors consists of a preoperative chemoradiation followed by surgical resection. High linear energy transfer (LET) radiation as, for example, C12 heavy-ion beam therapy theoretically offers biological advantages compared to high energy x-ray therapy as, for example, higher biological efficiency.

**Methods/Design:**

In the present prospective, single-armed, open pilot study performed at the Heidelberg Ion-Beam Therapy Center (HIT) in Heidelberg, the radiation treatment within the standard trimodal concept will be exchanged against C12 heavy-ion beam treatment and apply 39GyE in 13 single fractions in combination with a chemotherapy consisting of cisplatin and vinorelbine (local standard). The primary endpoint is feasibility and safety measured by the incidence of NCI-CTCAE grade 3/4 toxicity and/or discontinuation due to any reason. Secondary endpoint is the degree of regression in the histological specimen. The main inclusion criteria are histologically confirmed non-small-cell superior sulcus tumor, nodal disease stage ≤ N2, Karnofsky performance score ≥70%, patient age between 18 and 75 years as well as written informed consent. The main exclusion criteria include medical contraindications against elements of the trimodal treatment concept, PET confirmed nodal disease stage N3, stage IV disease, prior thoracic irradiation and decompensated diseases of the lung, cardio-vascular system, metabolism, hematopoietic and coagulation system and renal function. Furthermore, patients with implanted active medical devices without certification for ion-beam therapy are not allowed to take part in the study. Trial registration number: DRKS00006323 (www.drks.de).

## Background

Lung cancer is one of the most common cancers and accounts for 14% of all cancers in men and 13% in women [1]. The probability of lung cancer development during lifetime is 7.6% in men and 6.3% in women. In 2014 there will be approximately 224.210 new cases of lung and bronchial cancer in the United States. The estimated 5-year survival rate is 17% [1]. Superior sulcus tumors are a subtype of lung cancer, localized in the lung apex and commonly of non-small-cell histology. Treatment of choice is a preoperative chemoradiation followed by surgical resection [2]. Rusch et al. reported on preoperative radiotherapy applying 45Gy in combination with cisplatin and etoposide chemotherapy. The results showed 36% histological complete remissions in the surgical specimens. Acute adverse events > CTC °2 were primarily bone marrow suppressions. The trimodal treatment concept in Heidelberg is platin-based as well and applies cisplatin in combination with vinorelbine. The results of an en-block resection of superior sulcus tumors in Heidelberg were published by Pfannschmidt et al. in 2003 [3]. The colleagues did not find an impact on survival stratified by the radiotherapeutical concept, but a complete tumor resection was prognostic. However, most patients in their report did not receive chemotherapy in addition to irradiation.

In the setting of phase I/II studies colleagues in Japan evaluated heavy-ion therapy in stage I non-small-cell lung cancer (NSCLC). A dose escalation trial evaluated initially 47 patients treated in 18 fractions within 6 weeks (>86.4GyE) and later 34 other patients treated in 9 fractions within 3 weeks (72GyE) [4]. Grade 3 pneumonitis was reported in 3/81 patients, but all patients recovered completely and furthermore, the pneumonitis was not dose-limiting. The local control rates were 64% and 84%. The in-field recurrence rates of the initial series were dose-dependent. Applying doses >86.4GyE in 18 fractions or 72GyE in 9 fractions resulted in local control rates of 90% and 95%. Miyamoto et al. reported on heavy-ion therapy in 50 patients suffering from stage I NSCLC applying 72GyE in 9 fractions [5]. All patients were followed-up for at least 5 years or until death. The median follow-up time was 59.2 months and the local control rate 94.7%. No pulmonary toxicity > CTC °3 was reported. Comparable results were published by Hof et al. in 2007 on stereotactic radiosurgery using photon beams in stage I/II NSCLC [6]. Patients were treated with doses of 19 to 30Gy prescribed to the isocenter. The 12-, 24- and 36-months local control rates were 89.5%, 67.9% and 67.9%, and showed an advantage for higher doses. Furthermore, Grutters et al. reported in their meta-analysis on stage I NSCLC comparing photons, protons and heavy-ions a superiority of particles in comparison to conformal radiotherapy, especially regarding the survival probability; however, this survival advantage was comparable to stereotactic radiotherapy [7]. Furthermore, a trend to fewer side effects in particle therapy was seen, even though final conclusions could not be drawn.

Yamamoto et al. analyzed the surgical specimen in 4 patients having had surgical resection following heavy-ion therapy with 18 x 3.3 GyE [8]. Endpoints were tumor response and radiation induced changes within the lung tissue. In 2 patients no surviving tumor cells were found and in 2 other cases only scattered tumor cells. Furthermore, they described pulmonary fibrosis in the tissue surrounding the primary tumor, whereas the intensity decreased with increasing distance from the primary tumor site. Tissue having been irradiated with less than 15GyE did not show signs of pulmonary fibrosis. The authors concluded that their results demonstrated the efficacy and safety of heavy-ion therapy in NSCLC. Another team that evaluated radiographic changes of the lung and pleura following heavy-ion therapy were Nishimura and colleagues [9]. Their analysis showed a correlation of the V20 and V40 with the severity of the pulmonic changes. Furthermore, these changes occurred 3 months after the treatment and the maximum period was 6 months. In contrast, pleural changes occurred median 4 months after the initiation of heavy-ion therapy and correlated significantly with the PTV, V20 and V40. The effect on pulmonary function was evaluated by Kadono et al. [10]. The authors reported a <8% decrease in the forced expiratory volume in 1 second (FEV1) 6 and 12 months after heavy-ion therapy and rated this decrease in the FEV1 as not-severe. The impact of heavy-ion therapy on the migration and invasion of NSCLC-cells (cell lines A549 and EBC-1) was analyzed by Akino et al. [11]. The authors showed that heavy-ion therapy suppresses the metastatic potential in these cell lines.

## Methods and design

### Study design

This is a monocentric, single-armed, open, prospective pilot trial to generate basic data on hypofractionated heavy-ion therapy in superior sulcus tumors. Our intention is to test the safety and feasibility based on the incidence of NCI-CTCAE °3/4 side effects and/or discontinuation due to any reason. Objective of this pilot trial is the evaluation of the safety and feasibility of a preoperative hypofractionated radiation concept using active beam lines and C12-heavy-ions in the trimodal treatment approach of superior sulcus tumors. The in- and exclusion criteria are found in Table 1.

**Table 1:** **In- and exclusion criteria of the INKA study**

**Inclusion Criteria**	
Patients meeting all of the following criteria will be considered for admission to the trial:	
•	histological confirmed superior sulcus tumor (NSCLC)
•	maximal stage N2 in a FDG-PET-CT
•	age between 18 and 75 years
•	Karnofsky Performance Score ≥70
•	Written informed consent (must be available before enrolment in the trial)
**Exclusion Criteria**	
Patients presenting with any of the following criteria will not be included in the trial:	
•	refusal of the patients to take part in the study
•	medical contraindications against one of the parts in the trimodal concept
•	stage N3 disease in FDG-PET-CT
•	stage IV disease
•	previous radiotherapy to the thoracic region
•	Participation in another clinical study or observation period of competing trials, respectively
•	no capacity to consent
•	active medical devices, for which no approval for ion-therapy exists (i.e. cardiac pacemaker, defibrillator, …)
•	decompensated diseases of the lungs, cardio-pulmonal system, metabolism, hematopoetic system, coagulation system or renal function

### Endpoints

Primary endpoint is safety and feasibility based on the incidence of NCI-CTCAE °3/4 side effects and/or discontinuation due to any reason.

Secondary endpoint is the regression rate in the surgical specimen according to Junker et al. [12]: grade I: no or only little tumor regression; grade IIa: >10% vital tumor cells; grade IIb: <10% vital tumor cells; grade III: complete tumor regression. Furthermore, the metabolic tumor regression (PERCIST 1.0) according to Wahl et al. [13] will be evaluated as well as the morphologic tumor regression according to the RECIST criteria. Finally, quality of life will be evaluated.

### Ethical aspects

Approval by the Ethics Committee of the Medical Faculty of the University of Heidelberg (S-025/2013) and the Federal Office of Radiation Protection (BfS) (22463/2-2013-024) has been obtained. The trial is registered at the German Clinical Trials Register (www.drks.de; DRKS00006323).

### Radiation therapy/treatment planning and dose prescription

*Target definition:* gross tumor volume (GTV): the GTV is defined as the visible superior sulcus tumor and the PET positive lymph nodes. Clinical target volume (CTV): the CTV is defined as the GTV with a safety margin. An internal target volume (ITV) will be calculated on the basis of an individual 4D-planning CT.

*Organs at risk (OAR):* following OARs will be contoured: esophagus, lungs, brachial plexus, and spinal cord. The tolerance doses described by Emami et al. [14] (1/3 of the lungs ≤54% (α/β = 3Gy); spinal cord ≤ 92% (α/β = 2Gy)) or the recommendations of the quantitative analysis of normal tissue effects in the clinic (QUANTEC; lung V20 ≤ 30-35%, mean lung dose 20-23Gy [15]) should be respected. The brachial plexus and esophagus will be respected due to the prescribed total dose.

*Dose prescription:* 95% of the ITV should receive 39GyE in 13 fractions (5–6 fractions a week). BED2Gy: superior sulcus tumor (α/β = 10Gy): 42Gy.

*Chemotherapy*: according to the local standard chemotherapy regimen: cycle 1: cisplatin 80mg/m^2^ body surface & vinorelbine 25mg/m^2^ body surface; vinorelbine 25mg/m^2^ body surface on day 8; cycle 2 (concomitant to irradiation): cisplatin 80mg/m^2^ body surface & vinorelbine 15mg/m^2^ body surface; vinorelbine 15mg/m^2^ body surface on day 8.

**Figure 1:**
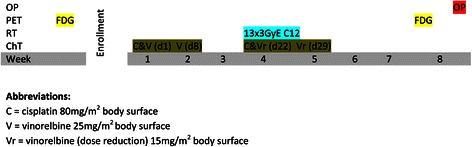
**Flowchart of the INKA study.**

*Course*: suitability check, enlightenment of the patient, informed consent, basic work-up including medical history and physical examination, QLQ-C30 and LC13 questionnaires, pulmonary function tests (FEV1); chemotherapy cycle 1, planning 4D-CT, heavy-ion therapy and concomitant chemotherapy cycle 2, restaging (FDG-PET-CT, QLQ-C30, LC13, pulmonary function test) followed by surgical resection in week 8, and follow-up examination including the final study visit as well as QLQ-C30 and LC13 in week 13-15. Flowcharts are found in Figure 1 and Table 2.

**Table 2:** **Overview of the INKA study**

Examination/point in time	Inclusion	Prior to chemotherapy	Prior to RT	During RT	Finish of RT	Week 8 preop.	Week 13-15	Month 6
Medical history	**x**		**x**		**x**	**x**	**x**	**x**
FeV1	**x**					**x**	**x**	**x**
Assessment of toxicity			**x**	**x**	**x**	**x**	**x**	**x**
Blood count	**x**	**x**	**x**	**x**	**x**	**x**	**x**	
FDG-PET-CT						**x**		
CT with i.v. contrast	**x**					**x**	**x**	**x**
Quality of life EORTC QLQ-C30, LC13		**x**	**x**			**x**	**x**	**x**

### Statistical considerations

#### Investigated populations

*ITT population*: the intention-to-treat (ITT) population includes all patients enrolled into the study. This population is defined according to the full analysis set (FAS) of the ICH E9 [16]. It is the basis for the primary statistical analysis.

*PP population*: the per-protocol (PP) population includes the all patients of the ITT population who have undergone the complete treatment and whose documentation is complete.

*Safety population*: the safety population includes all patients who were enrolled into the trial and who started the study treatment.

#### Study hypothesis and sample size

To use the data captured in this study in the most efficient way, in addition to descriptive analyses of all documented variables the results on tumor regression will be evaluated using inferential statistics. Within the confirmatory analysis, the null-hypothesis H_0_: p ≤ 0.20 will be tested, whereas p is the rate of complete tumor regression. For sample size calculation we assumed a rate p1 = 0.50. This choice regarding the null- and alternative-hypothesis results from a comparison with data from the literature (rate 0.36 reported by Rusch et al. 2007) and is justified by the increased validity of ion beam therapy (chosen rate *p*_1_ = 0.50 for the alternative-hypothesis) and the shorter period of time between chemoradiation and surgical resection in this study (approximately 2 weeks instead of 3–5 weeks, chosen rate *p*_1_ = 0.20 for the null-hypothesis). The null-hypothesis will be tested by an exact binominal test. A one-sided test at level α = 0.05 results in a power of 86% if 20 patients are included. This sample size is expected to be recruited within a reasonable period of time and allows a confirmatory statement on the efficacy of the chosen treatment concept in addition to the information gathered on safety and feasibility.

## Discussion

Lung cancer is one of the most common types of cancer with poor long-term survival rates. High complete response rates were found in histopathologic examinations following surgery with preoperative chemoradiation [2]. Even though a prior report by Pfannschmidt et al. did not show an impact of the chosen radiation technique on survival [3], recent studies demonstrated excellent results for local tumor control (>90%) after hypofractionated heavy-ion beam therapy in early stage NSCLC [4,5]. In addition, these excellent tumor control rates were associated with low treatment related adverse events [4,5,8]. For the dose calculations we assumed an α/β of 10Gy for sulcus superior tumors resulting in a BED2Gy of 42 Gy. To limit possible treatment-related adverse events, the recommendations by Emami et al. and QUANTEC were used [14,15].

Furthermore, high histopathological response rates (50% complete remissions) might be achieved considering the analysis by Yamamoto et al.; however, the number of analyzed specimens was small and higher doses (18 x 3.3GyE up to 16 x 4.5GyE) but without concurrent chemotherapy were applied [8]. Another analysis of heavy-ion beam therapy on the tumor cell migration and invasion suggested a suppression of the metastatic potential in the analyzed cell lines [11]. Recently, colleagues from Chiba reported on respiratory-gated carbon ion therapy in 34 patients treated for lung metastases from colorectal cancer (CRC) [17]. The carbon ion treatment was well tolerated (only Grade 1–2 adverse events) and achieved high local tumor control rates, even though Takeda et al. showed previously that lung metastases from CRC do not respond as well to stereotactic body radiation therapy [18]. Considering those reports we assume that the results of a preoperative heavy-ion beam therapy in combination with chemotherapy followed by surgical tumor resection might improve the outcome following high-linear energy transfer heavy-ion beam therapy in comparison to standard photon beam therapy for example due to biological effects allowing us to optimize the treatment results in the future while keeping treatment related adverse events minimal. Therefore, this prospective single-arm pilot trial is primarily aimed to evaluate the safety and feasibility of heavy-ion beam therapy for sulcus superior tumors in the trimodal setting. Secondary endpoint is the degree of regression in the histopathological specimen. In case of promising results, the information generated in the present trial will be used to plan a confirmatory study.

## Abbreviations

BED: Biological equivalent dose
BfS: Bundesamt für Strahenschutz (Federal Office of Radiation Protection)
C12: Carbon 12
CT: Computer tomography
CTCAE: Common toxicity criteria for adverse events
CTV: Clinical target volume
CRC: Colorectal cancer
FAS: Full analysis set
FDG: Fluorodeoxyglucose
FEV1: Forced Expiratory Volume in 1 Second
GTV: Gross tumor volume
Gy: Gray
GyE: Gray equivalent
HIT: Heidelberg Ion-Beam Therapy Center
ITT: Intention-to-treat
ITV: Internal target volume
LET: Linear energy transfer
NCI: National Cancer Institute
NSCLC: Non-small-cell lung cancer
OAR: Organ at risk
PERCIST: PET response criteria in solid tumors
PET: Positron emission tomography
PP: Per-protocol
PTV: Primary target volume
QLQ: Quality of life questionnaire
QUANTEC: Quantitative Analyses of Normal Tissue Effects in the Clinic
RECIST: Response Evaluation Criteria In Solid Tumors
V20: volume receiving 20Gy
V40: Volume receiving 40Gy
